# Evaluation of psychiatric conditions in asymptomatic outpatient clinic patients

**DOI:** 10.1371/journal.pone.0319168

**Published:** 2025-02-25

**Authors:** Osman Erinc, Soner Yesilyurt

**Affiliations:** 1 Department of Internal Medicine, Kanuni Sultan Suleyman Education and Research Hospital, Istanbul, Turkey,; 2 Department of Internal Medicine, Taksim Education and Research Hospital, Istanbul, Turkey; Taipei Veterans General Hospital, TAIWAN

## Abstract

**Introduction:**

A significant part of the internal medicine outpatient clinic burden consists of patients who are asymptomatic and intend to have routine check-up tests. In this study, we aimed to investigate the relationship between visit frequency within a year and the undiagnosed anxiety, depressive mood or obsessive-compulsive disorder.

**Methods:**

We included in our study 129 participants who applied for routine check-up tests to our hospital’s internal medicine outpatient clinic, without any complaint and known diseases. Individuals were divided into two groups: Group 1 comprised individuals who applied once a year, whereas Group 2 included those who applied more than once a year. Participants underwent routine blood testing, and their mental health was assessed with the Beck`s Depression Inventory (BDI), Beck`s Anxiety Inventory (BAI), and Vancouver Obsessinal Compulsive Inventory (VOCI).

**Results:**

66% of the 129 participants included in the study were female (n = 85/44, p < 0.001). When laboratory parameters were examined, no significant difference was found except serum vitamin D levels (14.5/19.8 µg/L, p = 0.024, respectively). BDI and BAI scores were statistically significantly higher in Group 2 (10/14, p = 0.032, 11/13.5, p = 0.027, respectively). There was no difference between the two groups in terms of VOCI scores.

**Conclusion:**

Asymptomatic patients who are visiting clinics for routine checkups constitute a significant part of the outpatient clinic workload. Assessing the mental health of patients who are attending frequently might be helpful in reducing this burden as well as in diagnosing and initiating treatment of undiagnosed underlying mental disorders. To ensure timely referrals of these patients to psychiatry, an adequate referral system and awareness of early signs of anxiety and depression among healthcare professionals are needed.

## Introduction

Internal medicine outpatient clinics have a crucial role in the prevention, diagnosis and treatment of many diseases that are common and have high morbidity and mortality, and are the most frequently consulted outpatient clinics [1].

Psychiatric disorders stand out as one of the major problems of the healthcare system all over the world [2,3]. The prevalence of mental illness detected in the “Turkey Mental Health Profile” study conducted at the national level is 17.2%, and 39% of those make their first visits to psychiatrists, while 33% to other specialties [4,5]. According to the data that was published by the World Health Organization in 2023, it is estimated that 5% of adults suffer from depression, and 280 million individuals being affected by this condition around the world, and each year more than 700,000 people die by suicide [6]. In low- and middle-income countries, approximately 75% of the patients who have depression or anxiety receive no therapy [7].

Due to the growing population globally and in our country, immigrant population transfer, which stands out as a global problem, and the decline in the ratio of health professionals to the general population, there are significant challenges in accessing health services. Furthermore, unnecessary and repetitive hospital visits, especially to specialty outpatient clinics, lead to overload in appointment systems and an increase in workload due to the burden they create. In our country, a total of 18% of people who request an appointment from outpatient clinics suffer from a waiting period exceeding 7 days [8,9].

While the global median of mental health professionals is 13 per 100.000 population, between 2 and 60 depends on income levels of countries [10], Turkey has approximately 228 physicians (including general practitioners, resident doctors, and specialized physicians) per 100,000 population, with a rate of about 6.7 psychiatrists [11]. On the other hand, the additional economic burden brought by unnecessary hospital visits and examinations to the healthcare system has negative effects on the general budgets of countries. The share transferred from the general budget to the health system in Turkey is 4.7%, and in the United States it stands out as approximately 17% [12]. While the share of the budget allocated for mental health in the total budget is 2.1% globally [10], this rate is less than 1% in a quarter of all countries [13]. Although data on the share of Turkey’s mental health budget in the total health budget is scarce, the low overall health expenditures indicate that few resources are allocated to mental health services [14]. Limited access to psychiatrists and constrained mental health budgets place additional importance on training general practitioners, internal medicine specialists and other clinicians to identify early signs of mental disorders. This approach can alleviate the strain on healthcare systems, minimize unnecessary diagnostic interventions, and facilitate timely referrals for specialized care.

Individuals with anxiety and depressive tendencies often have increased concerns about their health status [15]. Depression’s impact on health anxiety, cognitive function, and social factors contributes to frequent medical appointments. Also, these circumstances may lead to social withdrawal, resulting in individuals having insufficient emotional or social support resources. For some individuals, hospital visits may function as a different way of interaction or a method to fulfill these unmet psychosocial requirements [16]. Taking a thorough approach to addressing depression and anxiety can be important for reducing the number of hospital visits. The presence of accompanying psychological problems causes symptoms that cannot be medically explained by physical diseases for the clinician, resulting in a complex and mutually unsatisfactory relationship between the patient and clinician. This situation may cause a low level of satisfaction in health institutions, repeated hospital visits, and therefore a further increase in the already existing overload [17,18]. Despite patients applying to internal medicine outpatient clinics having medically unexplained symptoms, if they are not screened for psychiatric symptoms and not referred to psychiatric clinics as often as other subspecialties, this situation can lead to concerned patients not reaching their target clinicians, who could provide appropriate psychiatric care. Consequently, there is a critical need to evaluate the underlying psychiatric symptoms in patients visiting internal medicine outpatient clinics for routine exams. Addressing this gap could lead to diminishing unnecessary diagnostic interventions by identifying the psychological origins of medically unexplained symptoms, thereby lessening the diagnostic burden on clinicians and enhancing healthcare efficiency.

There are no particular limitations on the frequency of laboratory testing or health examinations in our country. A centralized hospital appointment system allows patients to arrange medical appointments from any specialties without the necessity of a referral system. This system does not impose any limitations on the frequency of visits. A significant portion of those who apply to the polyclinic are healthy individuals who do not have any complaints or known diseases and who request examinations to check their health status. To the best of our knowledge, our study is the first to evaluate underlying anxiety, depression, or obsessive-compulsive symptoms in patients visiting an internal medicine outpatient clinic.

## Methods

### Study design and setting

This study was conducted at a tertiary hospital that serves a population of approximately 1.5 million and currently has an existing referral system for psychiatric cases and includes a dedicated psychiatry department to address referrals. We included in our study 129 participants who applied for routine control examinations at our hospital’s internal medicine outpatient clinic between 01 January 2024 and 30 April 2024. The patients who had any diagnosed disease, were taking medication for any reason, or attended for any complaint and who underwent routine examinations for institutional and/or official reasons were excluded from the study. Also, patients exhibiting abnormal results in their last laboratory tests, as well as those with a history of psychiatric disease or prior psychiatric therapy, were not included in the study. These strict exclusion criteria aimed to obtain a homogeneous study population. Participants were split into two groups: Group 1 (n = 51) consisted of individuals who applied once a year, and Group 2 (n = 78) more than once a year. Participants in the study underwent routine blood testing, and their mental health was assessed via the clinician-administered Beck`s Depression Inventory (BDI), Beck`s Anxiety Inventory (BAI) and Vancouver Obsessinal Compulsive Inventory (VOCI).

### Beck's Depression Inventory

BDI includes 21 questions and measures the severity of depression. The items are graded on a 4-point scale from 0 to 3. The total score between 0–9 shows no or minimal depression, 10–18 mild, 19–29 moderate, and 30–63 severe depression [19].

### Beck's Anxiety Inventory

BAI consists of 21 items which are graded on a 4-point scale from 0 to 3. While 0–7 score indicates no or minimal anxiety, 8–15 mild anxiety, 16–25 moderate anxiety, and 25–63 score severe anxiety [20].

### Vancouver Obsessinal Compulsive Inventory

VOCI comprises 55 questions under six sub-categories of cleaning, checking, obsessive thoughts, hoarding, perfectionism and doubt. Answers to the questions are rated on a scale of from ‘0’ (not at all) to ‘4’ (very much). Total score is computed by summing the items [21].

### Ethical statement

All subjects provided written informed consent after receiving a thorough explanation of the study, both to participate in the study and to have data from their medical records. This study was conducted in accordance with the ethical principles outlined in the Declaration of Helsinki. The study protocol was approved by XXX Education and Research Hospital Ethics Committee with the date 27.12.2023 and number 173.

### Statistical analysis

The statistical software SPSS 26.0 was used for all of the analysis (Chicago, IL, IBM Corp.). The data distribution was checked using the Kolmogorov-Smirnov test and the results indicated that they do not have a normal distribution. Therefore, the Mann-Whitney U test was used for between-group comparisons. The variables were expressed as the median (quartile 25–75). Categorical parameters were indicated as percentages. Also, the correlation between frequency of visits to the hospital and psychiatric inventory scores was determined with the Spearman correlation test. The statistical significance cut-off value was deemed to be *p* < 0.05.

## Results

Table 1 includes the comprehensive data of study participants, revealing a predominance of female subjects, constituting 66% of the cohort (n (F/M) = 85/44, p < 0.001). The median ages of the groups are 37 and 39 years. When laboratory parameters were examined, no significant difference was found except in serum vitamin D levels between the groups. The median serum vitamin D level of the patients in Group 1 was found to be significantly lower than Group 2 (14.5/19.8 µg/L, respectively, p = 0.024).

**Table 1:** Demographic characteristics and laboratory findings of the groups.

	Group 1, n = 51	Group 2, n = 78	P
Age, years	37 (24–49)	39 (26–47)	.454
Female gender, n (%)	33 (64.7)	52 (66.7)	.819
Body mass index (kg/m2)	26.6 (22.9–29.2)	25.3 (21.5–28.9)	.370
Mean visit number/year	1	2.51 ± 0.752	**<0.001**
Laboratory findings			
White blood cell count, 10^3^/uL	6.9 (6.03–8.05)	6.87 (5.77–8.31)	.652
Hemoglobin, g/dL	13.7 (12.9–15.5)	13.2 (12.1–14.6)	.101
Platelet, 10^3^/uL	258 (236–297)	249 (212–280)	.127
CRP, mg/L	2.04 (0.99–4.68)	1.53 (0.72–3. 16)	.204
Blood glucose, mg/dL	88 (83–94.5)	91 (85.5–96)	.252
Hemoglobin A1c, %	5.6 (5.2–5.7)	5.4 (5. 1–5.7)	.390
Creatinine, mg/dL	0.69 (0.61–0.79)	0.69 (0.61–0.84)	.874
eGFR, mL/min/1.73 m^2^	113 (104–122)	112 (102–123)	.824
ALT, U/L	17.5 (13.7–32)	18 (13–27)	.397
Total bilirubin, mg/dL	0.51 (0.41–0.73)	0.57 (0.44–0.78)	.333
LDL, mg/dL	124 (98–150)	113 (92–151)	.217
HDL, mg/dL	56.5 (51–68.7)	58 (48–69.5)	.853
Tryglyceride, mg/dL	87 (61–138)	80 (60–147)	.604
Potassium, mmol/L	4.46 (4.22–4.64)	4.44 (4.2–4.63)	.801
Calcium, mg/dL	9.42 (9.21–9.68)	9.46 (9.2- 9.72)	.763
Magnesium, mg/dL	2 (1.9–2.1)	1.98 (1.88–2.11)	.525
Uric acid, mg/dL	4.9 (4–5.9)	5 (3.9–5.7)	.856
Vitamin D, µg/L	14.5 (11.4–23)	19.8 (13.5–25.9)	**.024**
Vitamin B12, µg/L	217 (166–345)	272 (174–358)	.337
Ferritin, µg/L	53.5 (22.4–91.6)	35 (18.6–67.9)	.103
TSH, mIU/L	1.79 (1.41–2.18)	1.82 (1.26–2.55)	.806

CRP: C reactive protein; eGFR: estimated glomerular filtration rate; ALT: alanin aminotransferase; LDL:, low density lipoprotein; TSH: tiroid stimulating hormone. Statistically significant values are indicated in bold. Data were presented as median (quartile 25–75)

As outlined in Table 2, BDI and BAI scores were statistically significantly higher in Group 2 (10/14, p = 0.032 for BDI; 11/13.5, p = 0.027 for BAI). There was no difference between the two groups in terms of VOCI scores (34/47, p = 0.077).

**Table 2:** Comparison of the BDI, BAI and VOCI scores between groups.

	Group 1, n = 51	Group 2, n = 78	*P*
BDI	10 (6–16)	14 (8.75–20)	**.032**
BAI	11 (6–14)	13.5 (7–23)	**.027**
VOCI	34 (19–62)	47 (26.2–79.7)	.077

BDI: Beck`s Depression Inventory; BAI: Beck`s Anxiety Inventory; VOCI: Vancouver Obsessinal Compulsive Inventory. Statistically significant values are indicated in bold. Data were presented as median (quartile 25–75).

The correlation analysis included all patients (N = 129), with a mean number of 1.91 ± 0.9 hospital visits within a year. A weak positive correlation was found between BDI, BAI, and the number of visits within a year (r = 0.177, p = 0.045; r = 0.194, p = 0.028). Concurrently, in terms of VOCI, no correlation was detected between groups (r = 0.157, p = 0.076) (Table 3).

**Table 3:** Correlation analysis between BDI, BAI and VOCI scores and number of visit within a year for all patients (N = 129).

		BDI	BAI	VOCI
Hospital visits/year	r	=0.177	=0.194	0.157
	p	**0.045**	**0.028**	0.076

BDI: Beck`s Depression Inventory; BAI: Beck`s Anxiety Inventory; VOCI: Vancouver Obsessinal Compulsive Inventory. Statistically significant values are indicated in bold.

## Discussion

In our study, we found that BDI and BAI scores were statistically significantly higher in Group 2 when compared to those who visited the internal medicine outpatient clinic once in a year (Group 1). There was no difference between the two groups in terms of VOCI. In addition, although there was a weak positive correlation between the number of applications in a year and BDI and BAI, no correlation was found with VOCI. Additionally, when both groups were compared in terms of laboratory data, a significant difference was found only in serum vitamin D levels. Patients in Group 1 had significantly lower serum vitamin D levels than those in Group 2.

In the literature, a gender effect was noted in the prevalence of common mental disorders, with women exhibiting higher rates of mood, anxiety, and obsessive-compulsive disorders, while men displayed elevated rates of substance use disorders [22–26]. In our study, the female gender was the majority, consistent with the literature.Considering the fact that the number of outpatient clinic applications and that especially mood and anxiety disorders are seen at a higher rate among the women, it can be thought that the evaluation of the underlying psychiatric symptoms should be done more carefully, especially in the female gender with recurrent outpatient clinic visits. Clinicians may adopt a gender-sensitive approach in their assessments, which could guide the development of targeted interventions to address these disparities effectively.

Gul A. et al. conducted a study with a total of 120 participants using the Short-form of Health Anxiety Inventory (HAE-SF), comparing two groups with those who had more than one visit in a year and those who had none. They found that those with a higher number of visits had higher health anxiety levels [27]. In our study, in addition to a positive correlation between the number of hospital visits and anxiety symptoms, consistent with this study, depression scores were also found to be high in those with a high number of applications.

In many previous studies, undiagnosed psychiatric disorders have been investigated in internal medicine clinics or in many different clinical situations in patients applied to the hospital for any reason. In the study of Huang Y. et al. published in 2019, consisting of 32,552 participants from the general population, the lifetime prevalence of depressive disorder was found to be 6.8%, anxiety disorder 7.6%, and obsessive-compulsive disorder 2.6% [28]. In contrast, studies focusing on clinical settings report significantly higher prevalence rates. For instance, in the study conducted by Yen Phi et al. in 2023 with 512 participants who visited a primary health care center for any reason, 15.8% of them had major depression or various levels of depressive disorders [29]. Gafaranga J.P. et al. found that the prevalence of depression was 45.7% in patients who applied to the internal medicine outpatient clinic, and 22.7% of them had thoughts of suicide [30]. Didden et al.‘s study in the internal medicine clinic revealed that 26% of patients had major depression, 16% had dysthymia, 9% had major depression in partial remission, and 13% had anxiety disorder [31]. The higher prevalence rates in these clinical settings compared to the general population may indicate the benefits of psychiatric symptom screening, particularly in internal medicine and primary care outpatient clinics. By integrating routine mental health screenings into the clinical workflows, healthcare providers can identify and address psychiatric symptoms earlier. Such integration could streamline diagnostic processes, reduce unnecessary diagnostic testing, and improve patient outcomes by ensuring timely referrals to mental health professionals.

Overutilization of medical procedures and tests is a significant problem in healthcare. The research conducted by Smart et al., encompassing 232 million participants, indicated that 14.4 million of 60.5 million individuals (23.8%) had expansive laboratory testing, possibly resulting in negligible benefit and possible detriment [32]. Beriault et al.`s study reported that 10–70% of laboratory tests may be redundant [33]. In our study, when hemogram and biochemical values were compared between the two groups, a significant difference was observed only in serum vitamin D levels. Vitamin D levels were found to be significantly higher in those who visited the outpatient clinic more frequently for routine check-ups. In light of the fact that no significant difference was observed between other examinations as the number of applications increased, it can be concluded that repeated applications and examinations bring an unnecessary workload and economic burden. From a resource allocation perspective, this study highlights the potential economic benefits of redirecting resources from redundant diagnostic tests toward mental health interventions.

This study has some limitations that should be noted. Firstly, since the design of our study was based on early detection of psychiatric symptoms rather than clinical diagnosis of psychiatric diseases, a diagnostic interview was not conducted by a psychiatrist. Without structured diagnostic interviews, such as the Structured Clinical Interview for DSM (SCID), the absence of formal psychiatric diagnoses can be considered a limitation of this study. Additionally, the observed differences in vitamin D levels between groups require cautious interpretation. Our study did not include a retrospective record scanning data on unprescribed treatments and supplementation regimens, which may have influenced these results. Importantly, our study’s lack of cost-effectiveness analysis constitutes an additional limitation. Analyzing the financial implications of unnecessary and repetitive healthcare applications, which include the costs for routine health check-ups, might provide important insights into the economic consequences of frequent healthcare utilization.

## Conclusion

Internal medicine outpatient clinics play a pivotal role in the early identification and management of undiagnosed psychiatric conditions, given their high patient volume and accessibility. Our study highlights an association between frequent outpatient visits and anxiety and depressive symptoms, emphasizing the need for an adequate referral system and awareness among health professions about early signs of anxiety and depression so that these patients can be timely referred to psychiatry. Addressing this unmet need can reduce outpatient clinics` workloads, improve patient satisfaction, and enhance overall healthcare efficiency.

To the best of our knowledge, although our study is the first to investigate the relationship between the number of visits to the internal medicine outpatient clinic and the three common psychiatric symptoms together, future studies with larger sample sizes, comprehensive diagnostic tools, and cost-effectiveness analyses are needed to further elucidate these relationships and optimize intervention strategies.

## Supporting information

S1 Dataset Patients’ dataset. (XLSX)
